# Diabetes and depression comorbidity and socio-economic status in low and middle income countries (LMICs): a mapping of the evidence

**DOI:** 10.1186/1744-8603-8-39

**Published:** 2012-11-26

**Authors:** Tiziana Leone, Ernestina Coast, Shilpa Narayanan, Ama de Graft Aikins

**Affiliations:** 1 LSE Health, LSE Houghton St, London, WC2A 2AE, UK; 2 Department of Social Policy, LSE Houghton St, London, WC2A 2AE, UK; 3 Appa Patwardhan Safai Wa Paryawaran Tantraniketan, Dehu Village, Pune, Maharashtra, 412109, India; 4 Regional Institute for Population Studies, University of Ghana, P. O. Box LG 96, Legon, Ghana

## Abstract

Non-communicable diseases account for more than 50% of deaths in adults aged 15–59 years in most low income countries. Depression and diabetes carry an enormous public health burden, making the identification of risk factors for these disorders an important strategy. While socio-economic inequalities in chronic diseases and their risk factors have been studied extensively in high-income countries, very few studies have investigated social inequalities in chronic disease risk factors in low or middle-income countries. Documenting chronic disease risk factors is important for understanding disease burdens in poorer countries and for targeting specific populations for the most effective interventions. The aim of this review is to systematically map the evidence for the association of socio-economic status with diabetes and depression comorbidity in low and middle income countries. The objective is to identify whether there is any evidence on the direction of the relationship: do co-morbidities have an impact on socio-economic status or vice versa and whether the prevalence of diabetes combined with depression is associated with socio-economic status factors within the general population. To date no other study has reviewed the evidence for the extent and nature of this relationship. By systematically mapping the evidence in the broader sense we can identify the policy and interventions implications of existing research, highlight the gaps in knowledge and suggest future research. Only 14 studies were found to analyse the associations between depression and diabetes comorbidity and socio-economic status. Studies show some evidence that the occurrence of depression among people with diabetes is associated with lower socio-economic status. The small evidence base that considers diabetes and depression in low and middle income countries is out of step with the scale of the burden of disease.

## Background

### Global burden of diabetes, depression and comorbidities

Diabetes causes 4.6 million deaths per year, accounting for 8.2% of global all-cause mortality, and it is estimated that 366 million adults have diabetes [1]. The global mortality burden of diabetes is not evenly distributed, with low and middle income countries carrying a disproportionate burden. It is projected that by 2030 around 82.5% of people with diabetes will live in developing countries [1]. The age distribution of adults with diabetes differs by country.

The occurrence of depression appears to be linked with the occurrence of diabetes. In 1684, Thomas Willis, the physician who first identified glycosuria as a sign of diabetes, suggested that diabetes resulted from ‘sadness or long sorrow and other depressions or disorders’ [2]. Further studies have demonstrated that a comorbid state of depression incrementally worsens health compared with depression alone [3]. According to the latest global burden of disease estimates unipolar depressive disorder are third in the ranking (65.5 mil DALY worldwide of which 26.5 in LICs). Unipolar depressive disorders are set to become the leading disease in 2030 with 6.3% of the overall burden and Diabetes the 10^th^ place with 2.3% as a percentage of the overall DALYs [4].

Comorbidity has various definitions and previous literature has highlighted the difficulty of defining it but in general, in medicine, it is usually considered as the presence of one or more disorders (in addition to a primary disease or disorder), or also the effect of such additional disorders or diseases [5]. In this study we look at the co-presence of diabetes and depression regardless of whether diabetes or depression is the primary disorder. The identification of co-morbidities is fundamental in order to understand whether the primary disorder or disease might either cause or affect the secondary one but also to understand any association between the two.

Studies have scrutinized the association of diabetes with depression and the bidirectional nature of this relationship; considering that depression may occur as a consequence of having diabetes, but may also be a risk factor for the onset of type 2 diabetes ^a^[6-8]. One study showed how there is a higher risk of mood and anxiety disorders among individuals with diabetes relative to those without, with an odds ratio for depression of 1.38 (95% CI 1.14-1.66) after adjusting for age and gender [9]. A meta-analysis concluded that the presence of diabetes doubles the odds of comorbid depression and the prevalence of comorbid depression among people with diabetes was 11% [10]. Estimates of depression prevalence among people with diabetes appear to vary by diabetes type and between lower and higher income countries, although the evidence base for lower income countries is much smaller than that for HICs [11]. A study conducted in 2007 which looked at depression worldwide using the WHO World Health Survey (WHS) found that 9.3% of people with depression were also with diabetes [3].

Two hypotheses attempt to explain the causal pathway between diabetes and depression. One hypothesis asserts that depression precedes type 2 diabetes, with depression occurring as a result of increased counter regulatory hormone release and action, alterations in glucose transport function and increased immuno-inflammatory activation. These physiologic alterations are thought to contribute to insulin resistance and beta islet cell dysfunction, leading to development of type 2 diabetes [12]. The second hypothesis is that depression in patients with both type 1 and type 2 diabetes results from chronic psychosocial stressors of having a chronic medical condition [13].

Evidence from HICs suggests that depression among people with diabetes is associated with socio-economic status [14], marital status [15], and physical activity and chronic somatic diseases [16]. Psychosocial factors may mediate the relation between SES and depression in people with diabetes, including social isolation or social support, coping styles, behaviour and job stress or strain [17,18]. Most studies show inverse social gradients, meaning that the risk is higher for people with lower SES [14-16,19]. However, the relationship may vary depending on the social and economic context of the country. In LICs, higher SES may be associated with higher levels of chronic disease risk factors in general [20] while the poor experience a double burden of infectious and chronic diseases according to the protracted polarised model of epidemiological transition [21].

In addition, the burden of risk factors for depression among people with diabetes in particular has been found to shift towards the less affluent in countries undergoing the epidemiologic transition where the cause of deaths shifts from infectious to non-infectious causes [22].

The aim of this systematic mapping is to identify the socio-economic factors associated with diabetes and depression as a comorbid condition exclusively in low and middle income countries.

## Methodology

We systematically mapped the evidence pertaining to poverty and depression-diabetes comorbidity in low and middle income countries. We searched 12 databases ^b^, selected for their coverage of the behavioural and social sciences, using combinations of keywords (diabetes, diabetes mellitus, chronic disease, depression, depressive disorder), and individual countries defined as low or middle income. The search included items written in English and with an abstract dated 1990–2011 and was completed in August 2011. Studies were also identified by hand-searching reference lists of reviews and articles found in the database search. We used broad search terms in order to include the widest literature possible in our mapping. This means that we not only identified items where the authors had measured or defined SES, but we also included items which considered variables often used as proxies for SES (e.g.: education, unemployment). The search was limited to studies published after 1989 and we identified 1747 relevant articles (Table 1).

**Table 1:** Studies (n = 14) included in the mapping

**Author**	**Year of publication**	**Sample size**	**Country of study**	**Association with SES**	**Recommended intervention**	**Clinical assessment**	**Analysis**
Agbir	2010	160	Nigeria	No significant association between patients with diabetes and depression and SES	Screen people with diabetes for depression especially those at “high risk” (e.g.: unmarried females); increase patient compliance to treatment, prevent complications, improve quality of life.	Clinical assessment of depression and blood glucose	Association of depression with newly diagnosed type 2 diabetes among adults
Eren	2008	108	Turkey	Negative correlation with SES (education)	Early detection and treatment of depression in people with diabetes.	Clinical assessment of depression and blood glucose	Impact of depression on diabetic quality of life.
James	2010	400	Nigeria	SES(Education, occupation, income)	Screening for depression among people with diabetes and management of depression to improve quality of life and reduce treatment costs	Questionnaire and blood glucose	Prevalence of depression and SES
Kilzieh	2008	2038	Syria	Comorbidity decreases with increasing SES	Deliver treatment for depression in primary care settings because access to mental health services are limited and stigmatised.	Questionnaire and diabetes self-reported	assess the comorbidity and correlates of depression in chronic diseases in a community
Mansour	2007	103, 103	Iraq	SES(Education, occupation, income)	None made	Questionnaire and blood glucose	Determine the prevalence of comorbid depression among sample of patients with type 2 diabetes mellitus. Control for social class
Mier	2008	200	Mexico and USA	Low education increases risk of depression	Depression screening among diabetic patients by family practice physicians.	Questionnaire and diabetes self-reported	prevalence and correlates of clinical depressive symptoms in Hispanics of Mexican origin with type 2 diabetes
Pan	2008	3285	China	Low education level and presence of co-morbidities associated with depressive symptoms	None made	Questionnaire and blood glucose	Association between insulin resistance and depressive symptoms
Raval	2010	300	India	Relationship between comorbidity and income and education unclear	None made	Questionnaire and blood glucose	Prevalence and determinants of depression in patients with established type 2 diabetes (T2DM)
Sevincok	2001	98	Turkey	Failed to find association between	None made	Clinical assessment and blood glucose	Assess association of socio-demographic variables for patients with and without comorbidity
Tellez-Zenteno	2002	189	Mexico	Higher risk of depression for lower SES	Screen for depression in all diabetic patients, so that early diagnosis and treatment can improve patient metabolic control and enhance patient quality of life.	Clinical assessment and blood glucose	Identify the prevalence and factors associated with depression in a group of patients with type 2 diabetes mellitus
Thaneerat	2009	250	Thailand	Association for co-morbid patients not clear	Early detection of depression among diabetic patients	self-reported questionnaire and blood glucose	To estimate the prevalence of depression, and poor glycemic control, and to determine the associated factors in outpatients with type-2 diabetes.
Yang	2009	148	China	Not clear	Early detection and treatment of depression in people with diabetes by community nurses and provision of social support.	Self-reported Questionnaire and diabetes self-reported	To examine levels of perceived social support and depression and to identify the related factors and
Yekta	2010	295	Iran	Lower educated higher risk of depression	None made	Self-reported questionnaire and blood glucose taken	To describe the prevalence of depression in patients attending a diabetes clinic determine the associated sociodemographic, behavioural and clinical factors.
Zhang CX	2008	304	China	Not clear	Identify source of patient stress; Advise on active coping styles; Mobilize more social support resources to reduce risk of depression in Type 2 diabetes.	Clinical assessment and blood glucose	To investigate association of psychosocial factors with anxiety and depressive symptoms in type 2 diabetes patients

Depression is responsible for the greatest proportion of disease burden associated with non-fatal health outcomes, accounting for approximately 12% of the total years lived with disability [4]. The evidence base and data for LICs are under-developed, but it is estimated that the average lifetime and 12-month prevalence estimates of major depression episodes was 11.1% and 5.9%, respectively, on the basis of data from eight LMICs [23].

Systematic mapping is a transparent technique for describing the research evidence on a topic. It not only allows us to take stock of the available research, but also to identify the gaps in the evidence base and how it might be developed [24]. The methodology for systematic mapping developed from work at the EPPI-Centre (Evidence for Policy and Practice Information and Co-ordinating Centre) and is being increasingly used in a range of social sciences [25-28]. The type of evidence and scope included in a systematic mapping is broader than that normally included in a systematic review, reflected in the breadth of the research questions. A systematic mapping can be much more inclusive [25-28] in its selection of studies than a systematic review can be. Inclusivity benefits the evidence base by assembling evidence in a systematic way. As a systematic mapping, rather than a systematic review, we have not assessed the quality of the included studies. This means that the evidence base that we have identified is not necessarily all of high quality.

### Inclusion and exclusion criteria

Abstracts were screened and items included if they addressed diabetes (type 2 or type 1 and 2 together to keep the disease type homogeneous), and depression as a comorbidity with diabetes, and SES. Studies were excluded if: they were conducted exclusively in HICs; did not address SES as a risk factor or consequence; the full-text was not in English; the study population was aged below 16 years; and, if there was no abstract.

### Defining and measuring SES

Our search strategy was deliberately inclusive in order to map the available evidence as widely as possible. Reflecting the different approaches to conceptualising socio-economic status are the indicators used to measure it. Rather than considering just one term such as “poverty”, we considered socio-economic status (SES) in general, including both individual-level (e.g.: education, occupation, income, household assets, place of residence, age, marital status, family type and social support) and household-, family- and community-level characteristics. Debates about conceptualising, defining and operationalizing socio-economic status are well-established and beyond the scope of this systematic mapping [29-34].

Characteristics of communities or neighbourhoods, such as the availability and accessibility of health services, infrastructure deprivation, prevailing attitudes towards health, levels of stress and social support, and environmental conditions, may influence general health outcomes [35]. The socio-economic status of a community may determine the educational, employment, and income opportunities of individuals and may also directly influence the social environment, although it is subject to the ‘ecological fallacy’ of assuming that all individuals in an area have similar characteristics [36].

### Defining and assessing depression and diabetes

Measurement of depression usually relies on structured interviews conducted by a professionally trained clinician or nursing staff using established criteria to identify a cluster of symptoms that may accompany depression (e.g.: loss of interest or pleasure in everyday activities, lack of appetite, fatigue, sleep disturbances, suicidal ideation). Most tools used to identify and rate the severity of depression rely on a multiple choice questionnaire, for example, The Hamilton Rating Scale for Depression (HRSD, HAM-D) the MINI questionnaire, the Beck Depression Inventory (BDI) [37]. It should be noted, however, that in many LICs lack the resources – both human and financial – to detect depression [38].

Similar problems of detection and diagnosis affect the valid measurement of diabetes in LICs Diabetes can be identified by either clinical blood glucose measurements, although some studies use “self-reported diagnosis” associated with diabetes. Self reports of diagnosis tend to be used in settings where glucose data are unavailable, and cannot distinguish between Type 1 and Type 2 diabetes. It is important to note that self-report might underestimate type 2 diabetes due to undiagnosed cases [11].

## Results

The search yielded 1,747 items, of which 1647 were excluded after abstract screening. Of the remaining 100 items, the full text via institutional (London School of Economics) access was only available for 63 studies, of which a further 11 were excluded because the full-text was not in English. Where full-text institutional access was not available, we used secondary databases (e.g.: Google Scholar) to try to retrieve full text of the remaining 37 items but none were available through this route. Most non-retrievable items were unpublished working papers with abstracts that were identified by the search, but not available electronically. The remaining 52 items were screened for inclusion on the basis of a review of their full text, after which a total of just 14 studies were selected for inclusion in the mapping. The main reason for exclusion at this stage was that an association between depression-diabetes comorbidity and SES was not sought or diabetes and depression cases were considered as separate diseases in two different populations rather than as a comorbidity (e.g.: diabetic patients with depression or viceversa) in a specific group of people.

### Description of included studies

All the 14 included studies were published post-2007, reflecting the nascent interest in depression-diabetes comorbidity in LICs. All of the included studies were cross sectional in design, and we did not identify any longitudinal or intervention studies, meaning that causal inference was not a possibility in our mapping. Just five studies [39-43] used a control case design to compare diabetic patients with and without depression. Three studies were community-based [43-45] while the rest where facility-based. It is important to separate facility- and community-based studies in order to take account of bias (Berkson’s bias), as barriers to accessing health care might bias results from facility based studies because they are more likely to include patients: from higher socio-economic strata; with more advanced disease; and, more likely to have another comorbid disorder than those in the general population [46].

Facility-based studies tended to have relatively small (at most n = 400) sample sizes and were carried out at tertiary hospitals, which in LICs might be more likely to cater for patients from higher socio-economic strata and with more advanced disease. This difference needs to be taken into account when making statements about true population differences, which might account for inconsistencies in association between socio-economic status and diabetes-depression comorbidity across studies. Studies which used control groups for comparison were not always clear about the characteristics of the control groups which could have potentially affected the effect of sample sizes on the overall results.

Of the facility-based (n = 11) studies, 4 studies had a control group, although they differed in control group selection [39-41,43,47]. A study from Nigeria recruited diabetic patients as cases and apparently healthy controls without a history of diabetes mellitus from local government staff of three local government areas [40]. A similar approach was used in a study from Iraq, which compared diabetic patients (case) with healthy controls drawn from hospital staff [41]. A study conducted in Turkey recruited diabetic patients and assessed them for presence of depression [39]. Finally, [42] assessed the prevalence of depression in Hispanics of Mexican origin with type 2 diabetes living on both sides of the Texas-Mexico border, recruiting people with type 2 diabetes from clinical settings which included hospitals and physicians’ offices on both sides of the border.

### Assessing socio-economic status

The operationalization and definition of SES in studies included in our mapping are heterogeneous. There is little or no discussion about the validity or reliability of the many difference measures of, and proxies for, SES. Studies that cautiously and robustly identify the presence of diabetes and depression comorbidity tend not to apply the same rigour to SES and its measurement. SES indicators in studies included in our mapping include indicators at a variety of scales, including individual and household.

Employment and education were the most frequently used variables to assess SES. Most studies included education as a proxy of SES [39-42,48-53]. Categorisations varied from literate-illiterate dichotomy [48,52] to years of education [40,50,51]. Employment was considered as a dichotomy (employed vs. unemployed) [40,48]. Three studies used income [40,41,52], and just one study used place of residence [52] to represent SES.

Finally, three studies used composite indicators of SES [44,53-55]. For example, a study from Syria assigned a score for SES based on work status, number of earning members within a household, household income, education level, item ownership and household density (number of individuals living in the household divided by the number of rooms) [44].

### Studying comorbidities: diabetes and depression

No study sought a causal relationship between SES and diabetes-depression comorbidity. The majority of studies considered the risk of, and risk factors for, depression in diabetic patients. Two community-based studies addressed diabetes and depression as a comorbidity – hereafter referred to as “direct diagnosis of comorbidity” [44,45]. The Kilzieh study [44] assessed the comorbidity of depression with other chronic diseases in a single Syrian city, using two stage, stratified cluster sampling, with a sample size of 2038. The second study, from China, was community based and conducted among people with type 2 diabetes (n = 148) and assessed the association between diabetes and depression comorbidity with SES [45].

The remaining studies looked at depression risk in patients with diabetes, hereafter referred to as “indirect diagnosis of comorbidity” because the comorbidity was assessed indirectly by considering the patient’s risk of depression. A notable finding, which helps to explain the lack of studies in the area of diabetes and depression comorbidity, is that in studies conducted at geriatric or diabetic clinics where patients came for treatment of chronic medical conditions, patients were often diagnosed with psychiatric comorbidity only as a result of going to the clinic. This suggests that there is a substantial burden of undiagnosed psychiatric disorders, including depression [48,50,51,53,54,56].

### Direct diagnosis of comorbidities and their relationship with SES

The study by Kilzieh [44] in Syria showed that depression comorbidity with any chronic disease decreased with higher SES (middle vs. low: OR = 0.41, 95% CI:0.22-0.78; high vs. low: OR = 0.52, 95% CI:0). An increase in comorbid depression in women with lower SES underlines the higher vulnerability of women to adverse mental health effects of lower SES. This relationship was not, however, confirmed in the relationship with education where a significant increase in depression comorbidity was reported in those with 1–9 years of education, which, according to the authors, may reflect ascertainment bias. That is, more educated individuals are more likely to seek medical care and consequently to be diagnosed with depression and chronic disease. This study also considered other proxies for SES, including the community-level proxy of place of residence, and found depression to be associated with disadvantaged neighbourhoods or “informal zones” (OR = 0.22, 95% CI:0.06-0.80) in the Kilzieh study [44]. Informal zones are areas in which houses were built without government approval, reflecting disadvantaged status.

Unemployment was significantly associated with depression in diabetic patients in the study by Yang [45]. At household levels, those with low income, less wealthy or those with fewer household assets were more likely to be depressed [44]. Finally, lower levels of social support were significantly associated with depression in the study by Yang [45] using a multidimensional scale of perceived social support.

### Indirect diagnosis of comorbidity and its relationship with SES

Socio-economic indicators at the individual level (e.g.: unemployment, education) were associated with depression in these studies that indirectly diagnosed depression-diabetes comorbidity [22,45,57].

A study from China found no significant difference in depressive symptoms between rural and urban dwellers (p = 0.129) [49]. This study was conducted in one rural county and two urban districts in two geographical locations of Beijing and Shanghai, which might account for the lack of an observed statistical difference because of the predominance of an urban population. However, this study did note a statistically significant association for women (but not men) between depressive symptoms and insulin resistance (OR 1.58, CI 1.14-2.18; P = 0.006) after adjusting for geographic location, residential region, age, educational level, smoking and drinking status, physical activity level, BMI category and comorbidity. By contrast, no significant association between depression comorbidity with place of residence was found in studies from Nigeria (p = 0.80) [48] and India (OR 0.76, CI 0.44-1.34, p = 0.35) [52]. Both studies were carried out in tertiary health care facilities, meaning that their samples tended to involve complicated cases, not necessarily representative of a true population difference.

Mansour et al.’s [41] Iraq study derived an indicator for “social class” based on an aggregate score of education, occupation and income. The control group had a higher social class than patients with diabetes, which could be explained by the recruitment of controls from the medical staff of the hospital.

Monthly income for diabetic patients was significantly and negatively correlated with depression scores in a study from Nigeria [40] (Pearson coefficient(r) = −0.207, p = 0.003). Similar findings are found from research in Iran which reported that depressed patients were poorer (64.1% vs. 52.4% had a low income level, p < 0.05) [53]. A decline in economic condition was significantly associated with depression among people with diabetes in a study from China using multiple regression analysis with adjustment for sex, age, marital status, educational level, income, employment, years since diagnosis of disease, and presence or absence of diabetes complications. (Beta 0.482, t value 2.059, p = 0.041 and partial correlation 0.132.) [54,55]. By contrast, in India depression comorbidity was significantly associated with high monthly income (OR 1.22, CI 1.03-1.41, P < 0.001) [52]. Finally, no significant association with monthly income and depression comorbidity was found in the Agbir study (P = 0.110) from Nigeria [48].

Drawing conclusions about the relationship between education and depression-diabetes comorbidity is difficult because of the highly heterogeneous ways in which education was conceptualised across the different studies, in part reflecting different education systems between countries. The majority of studies found no significant association between the depression comorbidity and education level for a range of countries including Nigeria (Chi-square 1.229, df = 1, P = 0.268) [48]; India [52] (literate vs. illiterate, OR 1.12; CI 0.93-1.46, P = 0.07). Studies that compared depressive and non-depressive groups also showed no significant difference in Nigeria (Chi-square = 0.705, P = 0.343) [40] and Turkey (t = 1.31, P > 0.05) [39] and (r = −0.07, P = 0.49) [43].

The remaining four studies all suggest that lower education is associated with depression among people with diabetes, including: education up to secondary was significantly associated with depression among people with diabetes (OR 2.39; CI 1.09-5.21, P = 0.029) [42]; and, people with diabetes who had <5 years of education were more likely to be depressed (OR 3.26, CI 1.57-6.80, p = 0.0004) [50]. Diabetic patients in Thailand with less than 12 years education were significantly more likely to be depressed (OR 2.33, 1.28-4.29, p < 0.01) [51]. Finally, depressed patients were less educated than non-depressed patients in an Iranian study (OR 4.20 CI 1.10-5.60; p < 0.0001) [53].

Considering the relationship between employment, as a proxy for SES, and the comorbidity the findings are equally mixed. Three out of five studies found no significant association between depression-diabetes comorbidity and employment, including studies from Malaysia (Chi-square = 0.429, p = 0.512) [47] and Nigeria (Chi square = 0.04, df = 1, P = 0.84) [48] and (Chi-square = 0.087, P = 0.445) [40]. Of the two studies, both from Mexico, which did find a relationship between employment status and depression-diabetes comorbidity both report the same direction: lower employment status was significantly associated with depression among people with diabetes [42,50].

Poorer levels of social (including family) support were significantly associated with depression among people with diabetes studies from Thailand (OR 4.10, CI 1.78-9.53, p < 0.01) [51] and Mexico (OR 2.79, CI 1.02-7.82, p = 0.02) [50]. Depressive symptoms were negatively correlated with subjective social support in China (Beta −0.162, t = −3.635, p = <0.000 and partial correlation −0.228) [54,55].

### Study limitations

There are limitations of our search strategy that have implications for the scope of included evidence. Firstly, we only included items with English abstracts, meaning that we are likely to have excluded from the mapping substantial research evidence which may be of relevance for this topic. We did, however, review the type and content of these non-English items on the basis of their title and abstract only. Among these studies, only three studies, all from Latin America, appeared to be relevant to our study. A study from Brazil concludes that among people with diabetes, higher education, low family and individual income predispose to symptoms of depression [58]. A study in Mexico concludes that among people with type 2 diabetes, significant differences between depressed and non-depressed participants were found in schooling, marriage type and occupation [59]. A study assessing trends in social and demographic inequalities in the prevalence of chronic diseases including diabetes and depression in Brazil [60] revealed a higher presence of chronic diseases in low socio-economic strata. The remaining non-English studies did not provide sufficient evidence in their abstract for us to describe them here [61-66]. Secondly, we excluded studies that consider diabetes and depression in low and middle income countries that did not explicitly include reference to SES or one of its proxies. Therefore, there are themes that are potentially linked with the pathways between SES and diabetes and depression that we have not explored in this mapping, which may further our understanding of the relationship. A third limitation is methodological. As a systematic mapping, rather than a systematic review, we have not assessed the quality of the included studies. This means that the evidence base that we have identified is not necessarily all of high quality. However, as a systematic mapping we set out to describe the available research in order to show the gaps in the literature and, by taking an inclusive approach to our search, we have identified studies of research and policy relevance. Fourth, studies that failed to find any significant relationship between depression and diabetes as a comorbidity and SES, might not be published, introducing the possibility of publication bias. However, this possibility is diminished by the fact that we did find, but did not include studies in which diabetes and depression comorbidity was not the principle focus of interest of many of the included studies and that depression was reported as the commonest psychiatric disorder while diabetes was one of the many chronic disorders in the populations under study [56]. Fifth, assessment of SES is heterogeneous, limiting statistical comparability. Sixth, we included studies that used self-reports of diabetes, meaning there is no differentiation between type 1 and type 2 diabetes. There are further limitations linked to our analysis which are due to the quality and quantity of the papers found. Given the heterogeneity of the SES indicators and the small number of studies found we could not perform either a meta-analysis or a causal chain analysis. Finally, our inability to electronically retrieve 37 full text items, identified on the basis of our abstract search, means that we were unable to review some potentially relevant items. The majority of these items were non-peer-reviewed items such as unpublished working papers. The inability to retrieve some items means that we have been unable to include some potentially relevant material in our mapping, limiting its breadth.

## Conclusions

Although the epidemiologic pattern of diabetes may differ according to the stage of health transition that a country is going through, the occurrence of depression among diabetic patients or independently seems to be associated with lower SES, through most of its variables amenable to measurement in epidemiologic studies. There exists an undiagnosed burden of psychiatric disorders in the population, with an increased risk among those from low socio-economic strata and the elderly.

Despite the differences in study quality and heterogeneity of measured socio-economic variables, there have been some recurrent associations. Depression was more likely to be present among the elderly, and among those with low family income, the non-professional/administrative class, those not currently employed and dependent, those living alone and with less social support. The relationship with education has been variable by country, showing a curvilinear gradient in the study from Syria [44], a significant association of low levels of education with depression in the studies from China [45,49]. Studies have also shown a higher prevalence of depression among women, [22,44,49,57] which could be influenced by sociocultural roles of women in these countries, including responsibilities at work and home, single parenthood, childcare, psychological attributes, or poor social support. Being married was a protective factor. Severity and duration of diabetes along with other comorbid conditions were more likely to be associated with depression.

More detailed research is needed to fully understand the relationship between SES and diabetes comorbid with depression. More generally, our mapping shows the need for research to address depression and diabetes together in LMICs. The size of the evidence base is out of step with the public health burden of this comorbidity. The proportion of the different components of SES contributing to this relationship might differ by the level of development of the economy, health systems and social support networks in these countries, the effect of one component mitigating the adverse effects of another. Understanding the multifaceted nature of socio-economic influences on health and the need to examine individual, system-level and community level factors and their relation to health behaviours and quality of care would be critical to the success of efforts at prevention.

Given the current epidemiological transition in LIMCs and with health systems struggling to cope with emerging non-communicable disease needs, this study highlights the strong need to develop further research in the field. This review indicates that there is some evidence for a consistent relation between SES and depression comorbid with diabetes, as well as with other chronic diseases. But the evidence is not strong enough to draw any sensible conclusions.

Most of the studies found in this mapping do not suggest solutions to the issues we highlighted. Future research could help to determine if the associations observed are consistent across diverse populations, which would be important to devise successful interventions to reduce disease burden in the most vulnerable populations. In addition, efficient social support could attenuate depressive symptoms in geriatric populations, in communities, and in particular, among diabetic patients.

However, we must bear in mind that social support is not always guaranteed for people with chronic conditions, especially in low income communities [67,68]; and especially when the symptoms of uncontrolled diabetes may evoke stigma [67]. We must also consider the fact that the presence of depression may exacerbate negative family and social responses to mental distress and mental illness such as neglect and abandonment [69].

In several low and middle income countries where there is limited access to specialty mental health services, as well as an associated stigma for utilizing these services, integrating these services with primary care providers by offering them training and support to treat depression would be an effective and efficient way of resource utilization. Furthermore, LICs are limited in their ability to offer appropriate NCD care at the primary care level because of socio-economic barriers, lack of insurance coverage, uncoordinated care, and shortage of physicians and specialist health workers. This is further limited by the lack of recognition of depression in many settings. Task shifting (of primary care duties from physicians to non-physician health care providers for management of chronic diseases) has worked for the provision of hypertension, diabetes and cardiovascular disease care in some LICs [70,71]. Its application, particularly at community level with community health nurses or lay health volunteers/workers may offer the best approach to reach individuals with co-morbid diabetes and depression.

## Endnotes

^a^Type 1 diabetes (previously known as insulin-dependent or childhood-onset diabetes) is characterized by a lack of insulin production. Type 2 diabetes (formerly called non-insulin-dependent or adult-onset diabetes) is caused by the body’s ineffective use of insulin. It often results from excess body weight and physical inactivity. (http://www.who.int/topics/diabetes_mellitus/en/).

^b^Pubmed, Embase, CabDirect, Psycinfo, Web of Science, Econlit, SocINDEX, Applied Social Sciences Index and Abstracts (ASSIA), International Bibliography of the Social Sciences (IBSS), Public Affairs Information Services International (PAIS) Global Health, PsycExtra.

## Competing interests

The authors declare that they have no competing interests.

## Authors’ contribution

TL and EC designed the study and the methodology. SN conducted the analysis and initiated the draft paper. TL wrote the overall paper. AGA commented and gave expert advice on the results and background. All authors read and approved the final manuscript.
